# Maturity-Onset Diabetes of the Young: Mutations, Physiological Consequences, and Treatment Options

**DOI:** 10.3390/jpm12111762

**Published:** 2022-10-25

**Authors:** Hazar Younis, Se Eun Ha, Brian G. Jorgensen, Arushi Verma, Seungil Ro

**Affiliations:** 1 Department of Physiology and Cell Biology, University of Nevada School of Medicine, Reno, NV 89557, USA; 2 Department of Pediatrics, Division of Pediatric Endocrinology, University of Nevada School of Medicine, Reno, NV 89557, USA; 3 RosVivo Therapeutics, Applied Research Facility, Reno, NV 89557, USA

**Keywords:** maturity-onset diabetes of the young (MODY), diabetes

## Abstract

Maturity-Onset Diabetes of the Young (MODY) is a rare form of diabetes which affects between 1% and 5% of diagnosed diabetes cases. Clinical characterizations of MODY include onset of diabetes at an early age (before the age of 30), autosomal dominant inheritance pattern, impaired glucose-induced secretion of insulin, and hyperglycemia. Presently, 14 MODY subtypes have been identified. Within these subtypes are several mutations which contribute to the different MODY phenotypes. Despite the identification of these 14 subtypes, MODY is often misdiagnosed as type 1 or type 2 diabetes mellitus due to an overlap in clinical features, high cost and limited availability of genetic testing, and unfamiliarity with MODY outside of the medical profession. The primary aim of this review is to investigate the genetic characterization of the MODY subtypes. Additionally, this review will elucidate the link between the genetics, function, and clinical manifestations of MODY in each of the 14 subtypes. In providing this knowledge, we hope to assist in the accurate diagnosis of MODY patients and, subsequently, in ensuring they receive appropriate treatment.

## 1. Introduction

Maturity-Onset Diabetes of the Young (MODY) is a monogenic type of diabetes. It is characterized by early onset of diabetes (before the age of 30), dominant autosomal inheritance, and hyperglycemia [1,2,3]. The mutated MODY genes often result from a single nucleotide substitution [4,5]. The involved genes are essential for proper pancreatic beta cell development and function. However, in mutated genes, glucose sensing and insulin secretion is impaired [1,3,6,7]. Mutations in hepatocyte nuclear factor 1–α (HNF1A), hepatocyte nuclear factor 1–β (HNF1B), hepatocyte nuclear factor 4–α (HNF4A), and glucokinase (GCK) account for most diagnosed MODY cases [2]. Furthermore, many of these genes are discovered to work in hierarchical fashion, assisting or hindering in the activation of other genes [8,9]. Even in the same MODY subtype, mutations can differ in terms of prevalence, clinical manifestations, and disease severity [7,10,11,12].

MODY is often misdiagnosed as type 1 (T1DM) or type 2 diabetes mellitus (T2DM) [2,13,14]. This can be avoided with adequate clinical knowledge and proper diagnostic techniques [15,16,17,18]. A simple key to distinguishing between T1DM or T2DM and MODY is age of diagnosis and parental history of diabetes [3,5,19,20,21]. One element complicating accurate diagnosis is low penetrance with certain subtypes of MODY, such as those with mutations in NEUROD1 [10,22]. A solid foundation in MODY subtypes and their genetic descriptions is critical for proper diagnosis and treatment of afflicted individuals.

## 2. Genetic Mutations and Functional Phenotypes in MODY Subtypes 

The genetic mutations, diabetic phenotypes, and treatments related to MODY1–14 subtypes are summarized in Table 1 and Table 2. The genetic mutations are well linked to hyperglycemia in all the subtypes. In addition, most mutations in the subtypes are also associated with hypoinsulinemia (Table 2). Many subtypes within type 2 diabetes have been shown to be effectively treated with sulfonylureas and insulin in case reports [23]. As previously published by Fajans and Brown, sulfonylurea-based drugs substantially augment the glucose-induced increases in plasma levels of insulin [24]. In addition, only MODY 3–5 have been reported to be treated with meglitinides [25], glucagon-like peptide-1 receptor agonists [26], sodium-glucose co-transporter-2 inhibitors [27], metformin [28], dipeptidyl peptidase-4 inhibitors [29], or repaglinide [30] in case reports (Table 2).

### 2.1. HNF4A-MODY1 (MODY1)

MODY1 corresponds with the HNF4A gene on chromosome 20. HNF4A encodes a transcription factor that belongs to the steroid/thyroid hormone receptor family. HNF4A is expressed primarily in the kidney, liver, gut, and pancreatic islets [4,6,131,132]. It plays a role in the metabolism of cholesterol, fatty acids, amino acids, and glucose. A previous study has reported that HNF4A regulates genes involved in glucose transport, glucose homeostasis, and genes essential to the functioning of pancreatic islet [6]. Yamagata et al. reported that mutations in HNF4A can cause a form of non-insulin-dependent (type 2) diabetes mellitus (maturity-onset diabetes of the young; gene named MODY1) [31]. MODY1 patients suffer from impairments in β-cell function with normal insulin sensitivity. Besides their β-cell associated phenotypes, MODY1 patients show impairments in glucagon and pancreatic polypeptide secretion, and decreased triglyceride and apolipoprotein biosynthesis [132,133]. 

There are several mutations that occur in the gene (Table 1), most of which are in the DNA binding, dimerization, and putative ligand domain. The first MODY1 mutation to be identified is Q268X, a nonsense mutation in which a stop codon is created at amino acid 268. The result is a truncated, partially functional protein. Evidence suggests that the DNA binding domain remains intact; however, the functionality of the putative ligand binding domain is compromised [4]. Insulin release rate as well as acute insulin response is decreased in diabetic patients with the HNF4A (Q268X) mutation. Interestingly, this remains true in non-diabetic patients who have the HNF4A (Q268X) mutation [6,132]. Further studies demonstrate that there is no dominant negative effect of Q268X. Instead, the phenotypes in different tissues could be explained by dose dependent HNF4 [6]. Mutant E276Q is the truncated, 40 kDa form and is characterized by loss of transcriptional activity as well as loss of DNA binding activity. It is possible that truncated HNF4A (E276Q) exhibits loss of function due to heterodimerization with the wildtype HNF4A protein. Thus, the amount of functional wildtype homodimers is diminished [6].

MODY1 was found to have a distinguished lipoprotein profile compared to other forms of diabetes. HNF4A is a key regulator of the ACAT2 promoter which is located in the liver and is responsible for catalyzing the formation of cholesteryl esters in hepatic VLDL assembly [4]. Many transcription factors are downstream of HNF4A. In the event of mutations in HNF4A, pancreatic transcription factors are upregulated in MODY1 cells. This may indicate the presence of a compensatory mechanism aimed to overcome haploinsufficiency caused by mutations in HNF4A. Putative HNF4A downstream target genes are more affected by the decrease in HNF4A levels if: (1) they contain multiple binding sites for HNF4A, (2) the distribution of binding sites is in proximity to the transcription start site of the target gene, and (3) they harbor fewer motifs for other transcription factors in their promoters. These molecular features influence the sensitivity of target genes and their ability to bind to HNF4A [6,132]. Patients with MODY1 are clinically characterized primarily by mild hyperglycemia [3,40]. Effective treatments for MODY1 patients are sulfonylureas and insulin [134].

### 2.2. GCK-MODY (MODY2)

Mutations in the central glycolytic enzyme glucokinase (GCK) gene can result in the development of MODY2 [1,2,135]. The structure of glucokinase consists of a large and a small domain separated by a deep opening where glucose binds [1,2,81,135]. GCK mutations have been found throughout the peptide structure that affect the enzymatic behavior but to different levels depending on the position and type of amino acid. Two regions that have a critical role during the conformational change observed in the active and inactive form are helices α13 and α5. So far, more than 620 GCK gene mutations have been reported in over 1400 patients with GCK-MODY. Most mutations are spread over ten exons of the gene which encode the pancreatic beta cell isoform of glucokinase [1,81,135]. MODY 2 is associated with heterozygous inactivating mutations in the GCK gene, which maps to chromosome 7 and spans 12 exons. The GCK gene encodes a glucokinase enzyme that plays a vital role in glucose metabolism. The kinetics differ from other hexokinases because of its lower affinity for glucose. GCK can have increased or decreased binding affinity to glucose depending on the type of mutation [2,33,136,137]. Missense, nonsense, frameshift, and splice site mutations have been reported, which are distributed throughout the 10 exons encoding the pancreatic β–cell isoform of the enzyme. As of now, there are no discovered mutational hot spots. Over 250 mutations have been reported in more than one family. In pancreatic β cells, the enzyme normally acts as a glucose sensor and regulates insulin secretion [33,137]. Activating mutations in the glucokinase gene (GCK) can cause hyperglycemia whereas inactivating mutations results in hypoglycemia [1,81,135]. Heterozygous inactivating mutations cause GCK–MODY. All inactivating mutations are associated with mild fasting hyperglycemia while other specific symptoms are missing. Therefore, GCK-MODY is likely to be underdiagnosed. Luckily, several GCK mutations have been genetically and functionally characterized which helped elucidate the link between mutation and function. Notably, the p. Gly68Val mutation causes structural conformational changes that lead to loss of function in the ability to bind glucose and ATP (Table 1) [34]. 

A single nucleotide mutation can induce a conformational change and affect the functionality of all components in the glucokinase enzymatic pathway. Inactivating and activating GCK mutations affect the mechanistic properties of this glucose sensor. With GCK mutations in particular, it is difficult to relate the mutations to clinical manifestations as it relates to insulin release and insulin resistance [1,33,81,136,137]. Patients with MODY2 with type 2 diabetes are clinically characterized by mild hyperglycemia (Table 1) [5]. Treatment of MODY2 is clinically unnecessary (Table 2) [130]. 

### 2.3. HNF1A-MODY 3

MODY3 is caused by mutations in the HNF1A gene. The protein product is localized to the liver, kidney, intestine, and pancreatic beta cells. It is thought to work in a hierarchical fashion, controlling the expression of insulin genes in mature beta cells. Additionally, HNF1A is proposed to control glucose transport genes (GLUT2) [9,138,139]. The two most common types of mutations associated with MODY3 are missense mutations which prevail in the dimerization and DNA binding domain of HNF1A and truncating mutations which are predominant in the transactivating domain [8,9,138]. Three novel mutations have been discovered (p.R171G, p.G245R, and p.R263H) (Table 1) [38] which reduce transcriptional activity and reduce nuclear localization. It is postulated that this occurs due to disruption of the three-dimensional structure of the protein product. A more common variant of HNF1A is p.S487N which results in loss-of-function and a more severe phenotype. Patients expressing these mutations oftentimes have transient neonatal hyperinsulinemia hypoglycemia, progressive hypoglycemia throughout childhood, gradual decrease in insulin secretion, and diagnosis with diabetes mellitus before the age of 25 [8,138,139].

### 2.4. PDX1-MODY (MODY4)

Genetic mutations in the pancreatic and duodenal homeobox 1 (PDX1) gene can contribute to MODY4. PDX1 is located on chromosome 7 and codes for an N-terminal transactivation domain, a homeodomain responsible for DNA binding and nuclear localization domain. It also codes for a conserved C–terminus which is often mutated in MODY4 [140,141]. PDX1 is a homeodomain-containing transcription factor, which regulates insulin gene expression, and is necessary for proper beta cell pancreatic development and function as well as the regulation of genes that code for glucagon, glucose transporter 2 (GLUT2), and other glucokinase enzymes [140,142,143,144,145]. PDX1 mutations can result in impaired expression of insulin and GLUT2 [145,146], permanent neonatal diabetes [89,147], and exocrine pancreatic insufficiency [146]. 

In vitro functional studies reveal that a single nucleotide substitution change of CCT to ACT at codon 33 resulted in a Pro to Thr substitution (P33T) in the IPF1 transactivation domain. P33T mutations were later determined to complicate pregnancies, where it is very likely for carriers of this mutation to develop gestational diabetes and give birth to underweight babies [140,148]. Patients with PDX1–MODY with type 2 diabetes have been shown to be effectively treated with metformin, dipeptidyl peptidase–4 inhibitors, and insulin, which are all options for treating individuals with MODY 4 in case reports [29,78,89].

### 2.5. HNF1B-MODY (MODY5)

MODY5 is caused by mutations in the hepatocyte nuclear factor-1β (HNF1B) gene; the HNF1B protein product is found in many organs and tissues including the lungs, liver, intestine, and pancreas [12,30]. The HNF1B functions in the development of renal nephrons and embryonic pancreas development. Mutation in the HNF1B gene can result in severe non-diabetic renal disease, glomerulocystic kidney disease, and hyperglycemia (Table 1) [42,43,44,45,46,47,48]. HNF1B is located on chromosome 17q12 and comprises nine coding exons. Fifty percent of patients with MODY5 have a whole gene deletion of HNF1B which is associated with severe pathology, elevated HbA1c, hyperglycemia, and decreased insulin secretion with progressive pancreatic β cell dysfunction (Table 2) [12,30,134].

Several mutations are associated with renal abnormalities and MODY5 in particular (Table 1). Molecular studies also reveal a frameshift mutation (c.C2304del) results in a truncated protein which affects the transactivated protein domain. This novel mutation is found to interact with, and have negative influence on, PKD2 and SOCS3, which play a role in renal abnormalities such as renal cysts and early diabetes onset [149]. Patients with HNF1B–MODY have been shown to be effectively treated with sulfonylurea [150], repaglinide [30], GLP-1 RA [93], and insulin in case reports (Table 2).

### 2.6. NEUROD1-MODY (MODY6)

MODY6 is caused by heterozygous mutations in the NEUROD1 gene, mapping to the chromosomal location 2q32. MODY6 is considered low penetrant. It is thought that the development of the disease is affected by genetic modifying factors such as epigenetics, environmental factors, and their interaction. NEUROD1 is a type II basic helix loop helix (bHLH) transcription factor with expression in pancreatic islet cells, intestine, and a subset of neurons in the central and peripheral nervous system. NEUROD1 is shown to interact with promoter regions and proteins. It can bind and activate the promoter of the sulfonylurea receptor 1 (SUR1), glucokinase (GCK), and PAX6. When these components are properly functioning together, normal glucose homeostasis is maintained [151,152,153]. 

Currently, 16 missense or truncated mutations of NEUROD1 have been genetically characterized, most of which are located in the bHLH or transactivation domain. Mutations in NEUROD1 manifest clinically in similar ways with a wide variety of affected functions. The mutations are associated with nephropathy, low C–peptide, high hbA1c, diabetic ketoacidosis, neurological abnormality, and in severe cases, renal failure [152,153,154]. Patients with MODY6 are clinically characterized by adult onset of the disease. Effective treatment of MODY6 is accomplished via insulin [130].

### 2.7. KLF11-MODY (MODY7)

KLF11 is an SP/Kruppel-like transcription factor which is mutated in MODY7. The function of Kruppel-like factor 11 is conserved in most species [51,155]. The KLF11 gene encodes for a transcriptional factor that regulates pancreatic β-cell function and impairment of insulin secretion (Table 2). Four missense mutations (R29Q, Q62R, T220M, and A347S) have been identified as associated with the development of diabetes [51,54]. KLF11 can also bind to GC-rich sites in the promoter region of the human insulin (INS) gene and inhibits the expression of INS. KLF11 binds and interacts with a host of epigenetic repressors including histone deacetylase 4 (HDAC4), Chromobox 5 (CBX5: HP1α), and Histone methyltransferase (HMT: SUV39H1), as well as a transcriptional repressor, SIN3A [15,17,51,53,156]. 

Interestingly, functional studies and co-immunoprecipitation assays reveal that KLF11 and PDX1 interact with one another to activate. This suggests that there is a hierarchical regulatory cascade for these two genes involved in development of MODY [53] One genetic variant of KLF11, A347S has been extensively studied and is revealed to affect transcriptional regulatory domain 3 (TRD3) [51]. With this variant, the binding is disrupted due to the mutation affecting the transcriptional regulatory domain 3 (TRD3) [15,17,51,54,156]. Patients with MODY7 are clinically characterized by pancreatic malignancy. Effective treatment of MODY7 is accomplished via insulin [130].

### 2.8. CEL-MODY (MODY8)

MODY8 is associated with mutations in the enzyme carboxyl ester lipase (CEL). It is also known as bile salt-dependent lipase or bile salt-stimulated lipase. It hydrolyzes fat-soluble vitamins, dietary fat, and cholesteryl esters in the duodenum. CEL is primarily expressed in pancreatic acinar cells and secondarily in lactating mammary glands [157]. CEL maps to the chromosomal location 9q34.3 and contains a carriable number of tandem repeats (VNTR) region that encodes a mucin-like protein tail. In MODY8, single-base pair deletions in the first VNTR repeat are the cause for disease state and pancreatic dysfunction, such as pancreatic lipomatosis and pancreatic cysts. This finding suggests that MODY8 can also be categorized as a protein misfolding disease. The C–terminal region of the CEL gene consists of an 11 amino acid repeat, trailed by the unique sequence KEAQMPAVIRF. Due to the variability in number of C-terminal repeats in the general population and the CEL gene’s general polymorphism, CEL is an excellent candidate gene for further studies [18,158]. 

Several mutations are identified as being associated with the development of diabetes (Table 1) [97]. Recently, two more novel single-base pair deletion CEL mutations have been identified, (c.168delT) and (c.1685delC), both of which have been associated with MODY8. Both occur on the VNTR segment of CEL, shift the reading frame, and prematurely truncate the splice variant at segment 13 of the VNTR sequence [158]. Upon examining the probands and their families, this mutation was associated with dominantly inherited insulin-dependent diabetes, pancreatic exocrine dysfunction, atrophic pancreas with lipomatosis, and pancreatic cysts [18,158]. Management of MODY8 includes dietary, oral antidiabetic, or insulin interventions (Table 2).

### 2.9. PAX4-MODY (MODY9)

MODY9 is caused by mutations in the gene encoding Paired box 4 (PAX4) transcription factor, which is essential for insulin production and pancreatic β-cell differentiation, development, and maintenance. PAX4 expression has also been linked to regulating pancreatic β–cell survival [159] and correlates with improved survival of pancreatic β–cells and a higher resistance to cytokine-induced apoptosis [160,161]. 

Several mutations have been identified as being associated with the development of nephrological diseases (Table 1). PAX4 (p.Q250del) was extensively studied and was found to have a disrupted splice site within exon 8. The result is a three-nucleotide deletion at position 250. When expressed in HEK 293 cells, both (p.Q250del) and wildtype PAX4 were found to normally translocate to the nucleus of β–cells. However, in the case of (p.Q250del), expression of the human insulin and glucagon promoter was inefficient compared to the wildtype [162]. Thus, the (p.Q250del) mutation impairs the PAX4 repressor functions on target-gene promoters (such as insulin and glucagon). It is also suggested that PAX4 mutations increase susceptibility to apoptosis in high glucose conditions [162]. Treatment of MODY9 is effectively managed by OHAs and insulin (Table 2) [130].

### 2.10. INS-MODY (MODY10)

MODY10 is caused by mutations to the insulin (INS) gene. The INS gene encodes preproinsulin that is targeted to and translocated across the endoplasmic reticulum membrane. The identification of mutations within INS shows an association with neonatal hypoglycemia (Table 1). Mutations in the INS gene can lead to defects in nuclear factor Kappa–light–chain–enhancer of activated B cells (NF-kB). Clinical manifestation of mutations in INS include decreased β–cells mass, gradual loss of insulin secretion, and variable-onset diabetes mellitus including neonatal diabetes (Table 2) [163]. Heterozygous missense mutations in INS can cause protein misfolding, protein aggregates, ER stress, and increased rate of apoptosis. Homozygous mutations of INS are associated with loss-of-function and decreased insulin biosynthesis by gene deletion. They lack translation initiation signaling and have altered mRNA stability (faster degradation). Certain INS mutations can lead to a more than 80% decrease in insulin production. Interestingly, homozygous mutations of INS represent 20% of total mutations in the gene [109,163,164,165]. Patients with MODY10 are clinically characterized by neonatal hypoglycemia (Table 1). Effective treatment of MODY10 is managed by insulin (Table 2) [130].

### 2.11. BLK-MODY (MODY11)

MODY11 is linked to heterozygous mutations in the tyrosine-protein kinase (BLK) gene belonging to the SRC proto-oncogene family. It encodes a tyrosine receptor protein which stimulates pancreatic beta cells to produce and secrete insulin. BLK is located on human chromosome 8p23–p22 and encodes a 505 length amino acid protein [112]. Unlike other subtypes of MODY, BLK–MODY is not highly penetrant; instead, it is described as having incomplete penetrance, meaning that several BLK mutations do not directly or necessarily cause diabetes (although they are still pathogenic). One such example of incomplete penetrance is the p.A71T mutation. It is a loss–of–function mutation which is shown to abolish the enhancing effect of BLK on insulin content and secretion from pancreatic beta cells [112]. The p.A71T loss–of–function mutation has a weak correlation with diabetes generally, although it may be “diabetogenic” in obese or overweight patients bearing the mutation [112]. MODY11 is effectively treated by OHAs and insulin (Table 2) [130]. 

### 2.12. ABCC8-MODY (MODY12)

MODY12 is caused by heterozygous mutations in the ATP binding cassette subfamily C member 8 (ABCC8) gene. ABCC8 encodes sulfonyl-urea receptor 1 (SUR1), a subunit of ATP-sensitive channels found in pancreatic beta cell membranes. ABCC8 is highly correlated with secretion of insulin and regulating blood glucose levels. Notably, mutations in ABCC8 are linked to congenital hyperinsulinism [66,120,150,166]. Patients with ABCC8-MODY can sometimes be underweight and present with hypoglycemia although other patients can have opposite complications [114]. 

Over 125 ABCC8 mutations have been found; 28 of these mutations have been extensively studied and summarized thus far, 25 of which are missense mutations, 1 a splicing mutation, 1 a small deletion, and 1 a small insertion [65]. The missense mutation p.Gly826Asp leads to increased fasting plasma glucose levels, hyperglycemia, and increased ABCC8 protein concentration (Table 1). Mutations in ABCC8 directly affect insulin secretion and ATP-sensitive potassium channel dysfunction (Table 2). Abnormal insulin secretion leads to glucose metabolism disorder [65]. ABCC8 mutations also lead to increased ABCC8 protein concentrations due to inhibition of the protein degradation pathway. Mutation p.Gly826Asp specifically has been shown to block both the ubiquitination and autophagy lysosome degradation pathway [65]. Patients with MODY12 are clinically characterized by renal diabetes (Table 1). MODY12 is effectively treated with insulin and sulfonylureas (Table 2) [130].

### 2.13. KCNJ11-MODY (MODY13)

MODY13 is caused by heterozygous mutations in the potassium inwardly rectifying channel subfamily J member 11 gene. KCNJ11 is mapped to chromosome 11p15.1. This gene encodes for pancreatic beta cell inwardly rectifier (BIR). More importantly, it encodes the Kir6.2 subunit of the adenosine triphosphate sensitive potassium (KATP) channel, which plays a key role in insulin secretion. Several mutations in the KCNJ11 gene are associated with renal diabetes, hyperinsulinemic hyperglycemia and neonatal diabetic phenotypes (Table 1 and Table 2) [70]. 

Mutation p.E322K has severe effects and is associated with permanent neonatal diabetes mellitus and causes convulsions due to extreme hypoglycemia. Neurological abnormalities (such as developmental delays, autism, ADHD, sleep disorders, and seizures) are also reported in p.E322K and other KCNJ11 mutations (Table 1). Not all mutations show severe clinical manifestations. Mutation p.E227G is associated with penetrance of hyperglycemia in family members with the mutation. It is also linked with transient neonatal diabetes [70]. Patients with MODY13 are clinically characterized by renal diabetes, hyperinsulinemic hypoglycemia, and neonatal diabetes (Table 1). MODY13 is effectively treated with sulfonylureas (Table 2) [130].

### 2.14. APPL-MODY (MODY14)

MODY14 is caused by mutations in the adaptive protein phosphotyrosine interacting with PH domain and leucine zipper 1 (APPL1) gene. APPL1 is responsible for regulating cell proliferation and interaction between adiponectin and insulin signaling pathways. In tissues where APPL1 mutated proteins are highly expressed, premature apoptosis is more likely to occur. Overexpression also causes dysmorphic phenotypes and developmental delays [77]. 

APPL1 binds to AKT2 (RAC-beta serine/threonine-protein kinase or AKT serine/threonine kinase 2). AKT2 is a key molecule in the insulin signaling pathway. APPL1 can enhance insulin-induced AKT2 activation and downstream signaling in the liver, skeletal muscle, adipocytes, and endothelium. APPL1 potentiates the inhibitory effect of insulin on hepatic gluconeogenesis through activation of AKT protein kinase [77]. The p.Leu552Ter is a missense mutation which results in premature truncation and a deletion of most of the phosophytyrosine binding (PTB) domain, resulting in diminished insulin-induced downstream signaling in target tissues (Table 2) [77]. Patients with MODY14 are clinically characterized by Wolfram or DIDMOAD syndrome (Table 1). MODY14 is effectively treated with OHAs or insulin (Table 2) [130].

## 3. Conclusions

MODY is a rare form of diabetes that is difficult both to diagnose and to characterize. Clinical manifestations of the disease resemble those of T1DM and T2DM. This is also complicated by subtypes of MODY which are of low penetrance. The subtypes of MODY are caused by specific mutations in various genes with widely different functions. These various mutated genes can exert repressive or activating influence on transcriptional pathways, and thus can influence the function of target tissues. Genetic mutations in all MODY subtypes are well associated with diabetic phenotypes (hyperglycemia and hypoinsulinemia). However, recently, Laver et al. reported that variants in BLK, PAX4, and KLF11 are not causative of MODY [166]. Moreover, some subtypes are not only linked with diabetes, but also with pancreatic, renal, or neurological pathologies. More functional studies and characterizations (e.g., β cell function and insulin resistance) for each mutated MODY subtype will offer pathogenic mechanisms, which will lead to the development of diagnostic tools and therapeutic approaches for a variety of MODY subtypes. 

## Figures and Tables

**Table 1:** Genetic mutations and clinical significance in MODY subtypes.

Subtype	Gene Name	Gene Symbol	Locus	Mutation		Clinical Significance	Ref.
				DNA	Amino Acid		
MODY 1	Hepatocyte nuclear factor 4α	HNF4A	20q13.12	763C>T 826G>C	Gln255Ter Glu276Gln	Mild hyperglycemia	[3,31]
MODY 2	Glucokinase	GCK	7p13	89T>C 203G>T 748C>T 905T>A 1363G>C	Leu30Pro Gly68Val Arg250Cys Val302Glu Ala455Leu	Mild hyperglycemia	[5,32,33,34,35,36]
MODY 3	Hepatocyte nuclear factor 1α	HNF1A	12q24.31	511C>G 733G>C 788G>A 1460G>A	Arg171Gly Gly245Arg Arg263His Ser487Asn	Neonatal hypoglycemia	[19,20,37,38]
MODY 4	Insulin promoter factor 1	PDX1	13q12.2	176A>T 188del 533A>G 590G>A 947G>A	Gln59Leu Pro63fs^* ^ Glu178Gly Arg197His Gly316Asp	Neonatal hypoglycemia	[10,21,39,40,41]
MODY 5	Hepatocyte nuclear factor 1β	HNF1B	17q12	335G>C 406C>G 490A>C 494G>A 884G>A	Arg112Pro Gln136Glu Lys164Gln Arg165His Arg295His	Renal disease Diabetic ketosis Glomerulocystic kidney disease	[22,42,43,44,45,46,47,48]
MODY 6	Neuronal differentiation 1	NEUROD1	2q31.3	34G>C 590C>A	Gly12Arg Pro197His	Adult onset (mid-20s)	[49,50]
MODY 7	KLF transcription factor 11	KLF11	2p25.1	86G>A 185A>G 659C>T 1039G>T	Arg29Gln Gln62Arg Thr220Met Ala347Ser	Pancreatic malignancy	[51,52,53,54]
MODY 8	Carboxyl ester lipase	CEL	9q34.13	1402G>A 1454T>C	Ala468Thr Ile485Thr	Adult onset (36 years) Hypoglycemia	[18,55,56]
MODY 9	Paired box 4	PAX4	7q32.1	385C>T 514C>T 539G>A	Arg129Trp Arg172Trp Ser180Asn	Nephrological diseases	[57,58]
MODY 10	Insulin	INS	11p15.5	25C>T 130G>A 137G>A 155C>A 290C>G	Pro9Ser Gly44Arg Arg46Gln Pro52His Thr97Ser	Neonatal hypoglycemia	[59,60,61]
MODY 11	BLK proto-oncogene, Src family tyrosine kinase	BLK	8p23.1	41C>T 116C>T 164A>G 177C>G 187G>A 311G>T 391C>T 713G>A	Pro14Leu Pro39Leu His55Arg Asp59Glu Val63Met Arg104Ile Arg131Trp Arg238Gln	Neonatal hypoglycemia obesity	[62,63,64]
MODY 12	ATP binding cassette subfamily C member	ABCC8	11p15.1	502C>T 2477G>A 3158G>A 3202T>A 4148G>C 4500C4A	Arg168Cys Gly826Asp Ser1053Asn Phe1068Ile Gly1383Ala Ser1500Arg	Renal diabetes	[65,66,67,68]
MODY 13	Potassium inwardly rectifying channel subfamily J member 11	KCNJ11	11p15.1	67A>G 679G>A 808C>G 902G>A 964G>A 973C>A 1034C>T 1040G>A	Lys23Glu Glu227 Lys Leu270Val Arg301His Glu322Lys Arg325Ser Thr345Met Arg347His	Renal diabetes Hyperinsulinemic hypoglycemia Neonatal diabetes	[69,70,71,72,73]
MODY 14	Leucine zipper containing 1	APPL1	3p14.3	280G>A 1655T>A 1926A>G	Asp94Asn Leu552Ter Ile642Met	Wolfram or DIDMOAD syndrome	[74,75,76,77]

*Fs, frameshift.

**Table 2:** Pathologies, phenotypes, and treatments of MODY subtypes.

Subtype	Pathophysiology	Phenotype		Treatment	Ref.
		Glucose	Insulin		
MODY 1	Progressive decrease in insulin secretion β-cell dysfunction Worsening of glucose control Low levels of apolipoproteins and triglycerides Neonatal hypoglycemia	↑	↓	Sulfonylureas, insulin	[78]
MODY 2	Higher glucose threshold for insulin release Glucose-sensing defects β-cell dysfunction Mild hyperglycemia (HbA1c 7.3–7.5%)	↑	↓	Treatment is unnecessary	[78,79,80,81,82]
MODY 3	Insufficient glucose-mediated insulin secretion β-cell dysfunction Low glucose renal threshold	↑	↓	Sulphonylureas (additional meglitinides, GLP-1 RA, SGLT-2 inhibitors), insulin	[25,82,83,84,85,86,87,88]
MODY 4	β-cell dysfunction Impaired glucose-mediated insulin secretion Mild form of diabetes Overweight/obesity in some patients	↑	NA	Sulphonylureas, insulin, metformin, dipeptidyl peptidase-4 inhibitors	[29,78,89]
MODY 5	β-cell dysfunction Decreased insulin secretion with progressive worsening of glucose control Genitourinary malformations	↑	NA	Sulfonylurea, repaglinide, GLP-1 RA, insulin	[45,46,78,90,91,92,93]
MODY 6	β-cell dysfunction Insulinopenia or insulin resistance Different degrees of hyperglycemia	**↑**	NA	Insulin	[94,95,96]
MODY 7	Decreased glucose sensitivity of β-cells Decreased sensitivity to insulin Mild hyperglycemia	**↑**	↓	Insulin	[51,53,97,98,99]
MODY 8	Impaired endocrine Exocrine pancreatic insufficiency (dysfunction of the mature acinar cell)	**↑**	↓	OHAs or insulin	[78,100,101,102,103]
MODY 9	β-cell dysfunction Progressive hyperglycemia Occurrences of ketoacidosis	**↑**	NA	OHAs or insulin	[96,97,104,105]
MODY 10	Hyperglycemia β-cell dysfunction	**↑**	↓	Insulin	[61,106,107,108,109]
MODY 11	Hyperglycemia β-cell dysfunction Affected insulin secretion	**↑**	NA	OHAs or insulin	[62,110,111,112,113]
MODY 12	Impaired insulin secretion ATP-sensitive potassium channel dysfunction	**↑**	↓	Insulin, sulfonylureas	[67,78,114,115,116,117,118,119,120]
MODY 13	Impaired insulin secretion ATP-sensitive potassium channel dysfunction	**↑**	NA	Sulfonylureas	[121,122,123,124,125,126,127,128,129]
MODY 14	Impaired glucose-mediated insulin secretion Hyperglycemia Reduced beta cell survival	**↑**	↓	OHAs or insulin	[74,75,76,77,130]

**↑**, hyperglycemia or hyperinsulinemia; ↓, hypoinsulinemia; N/A, not available; OHAs, oral anti-hypoglycemic agents; GLP-1 RA, glucagon-like peptide-1 receptor agonist; SGLT-2, sodium-glucose cotransporter-2.

## Data Availability

Not applicable.
